# Preface

**DOI:** 10.1098/rsta.2008.0046

**Published:** 2008-04-11

**Authors:** Kelly H. Zou, Aiyi Liu

**Affiliations:** 1 Harvard Medical School180 Longwood Avenue, Boston, MA 02115, USA; 2 National Institute of Child Health and Human Development6100 Executive Boulevard, Rockville, MD 20852, USA

Sophisticated diagnostic modalities such as a large number of biomarkers and advanced imaging tools have become common nowadays and pose challenges in analysis, modelling and interpretation of the high-dimensional data these modalities yield. These challenges motivate researchers to develop complex mathematical and statistical models using innovative and efficient analytic methods. To complicate the issue, omitted variables and missing data are frequently encountered and need to be dealt with carefully. In various therapeutic areas, the high-dimensional data require new visualization and analytic tools. The overall purpose of this Theme Issue is to present newly developed innovative mathematical and statistical modelling and analytic approaches for complex diagnostic and therapeutic data. The social impact of this body of work is to improve therapeutic outcomes by evaluating the accuracy, reliability in medical diagnoses and treatments by analysing complex biomarkers and multivariate data.

There have been a number of recent developments in the mathematical and statistical modelling of the processes generating clinical data, as well as improved methods of assessing whether deviations from the ‘ideal’ assumptions, which often arise in practice, could seriously affect the scientific conclusions. For example, clinical data pertaining to biomarkers and assays, missing data and high-dimensional medical image analyses are at the forefront of current medical treatments. Traditionally, almost all statistical methods assume that the data satisfy a few important assumptions and contain information about the variables related to the response under investigation. In practice, however, most datasets are imperfect, i.e. some may not be possible to obtain complete measurements for all potentially relevant variables. Inevitably, critics of a study point out these deviations from a ‘perfect’ study to cast doubt about the main findings. Sensitivity analysis has become an established method for assessing whether a ‘flaw’ in an otherwise competent study could ‘explain’ or ‘mask’ a scientifically valid association. The article by Yu & Gastwirth (2008) develops sensitivity analysis for the stratified data analysed by one of the most frequently used statistical techniques, the Cochran–Mantel–Haenszel test, and illustrates its use on real data.

Concerning the policies on a national level, the US National Institutes of Health reported on medical errors (‘To err is human: building a safer health system’), while the UK Department of Health report ‘An organisation with a memory’ emphasized the need for a structured response to the previously less-recognized problem of medical errors in healthcare (Department of Health 2000; Kohn *et al*. 2000). However, the health care delivery system and its structural cause-and-effect relationships have become increasingly complex and more difficult to characterize. Modern mathematical techniques of risk management, usually applied to high-hazard industrial settings, may be applied to this problem with a goal of reducing medical errors and improving patient safety. The article by DiDomenico *et al*. (2008) examines safety monitoring methods using Bayesian sequential methods.

The authors of the subsequent 12 articles consider such quantitative problems of evaluating the performances of medical diagnoses and therapies. The tools used for conducting the analyses include both mathematics (i.e. partial differential equations and geometric flow methods) and statistics (i.e. Bayesian methods, multivariate and high-dimensional data analysis, expectation maximization, Monte Carlo simulation, sequential analysis and missing data imputation). To name a few more, Shiu & Gatsonis (2008) examine the behaviour of the pair of positive and negative predictive values of a test as the threshold for test positivity varies. By studying the resulting predictive receiver operating characteristic curve, the authors demonstrate complex patterns of covariation of these two measures of predictive performance. O'Malley (2008) uses Bayesian predictive inference to construct a precision profile for an immunoassay by developing a novel measure of precision based on the accuracy with which an assay could infer the concentration in a hypothetical new sample. Based on multivariate multilevel continuous data with ignorable non-response, Yucel (2008) develops pragmatic computational tools to be used in drawing inference via multiple imputation in multilevel applications with missing values.

By developing these new methods in this Theme Issue, the authors and the editors hope that they will ultimately benefit clinical and scientific readers.
